# Dynamic principles of the microbiome and the bovine vagina: a review

**DOI:** 10.3389/fmicb.2024.1434498

**Published:** 2024-12-05

**Authors:** Nicholas Wege Dias, Rebecca Poole, Dallas R. Soffa, Kyle Joel Hickman Brown

**Affiliations:** ^1^Department of Animal Sciences & Industry, Kansas State University, Manhattan, KS, United States; ^2^Department of Animal Science, Texas A and M University, College Station, TX, United States

**Keywords:** microbiome review, bovine vaginal microbiome, bovine fertility, reproductive microbiome, microbiome ecology

## Abstract

The role of microbes inhabiting various body sites in supporting host physiology and health is substantial, and recent advancements in DNA sequencing technology have facilitated a more in-depth understanding of these microbial contributions. The influence of microbiota within a given organ can be broadly categorized as having two main functions: (1) promoting organ homeostasis and (2) creating conditions that inhibit the growth of pathogenic microorganisms, thereby protecting the host from diseases. In livestock production, numerous phenotypes critical to industry outcomes are affected by the microbiome, which has sparked considerable academic interest in recent years. This review aims to analyze the extensive data available on the microbiomes of humans and other mammalian species, examining microbiome ecology to elucidate principles that may assist in interpreting data on livestock microbiomes. Additionally, the review will discuss techniques available for investigating various microbiome aspects and will examine existing data on the reproductive microbiome, with a particular focus on the bovine vaginal microbiome.

## Introduction

In recent decades, reproductive technologies have advanced significantly, enabling faster and more effective genetic improvements in livestock. However, infertility remains a leading cause of economic losses, with the beef industry alone experiencing annual costs estimated to exceed $2.8 billion due to infertility (Lamb et al., 2014). The relatively new field of the reproductive microbiome in livestock, particularly regarding its connection to fertility, has generated growing interest among animal science researchers. Microorganisms present in specific body sites can have profound effects on phenotypes that impact economically relevant traits in livestock. However, the microbiome’s complexity, with numerous microbial species often inhabiting a single organ, complicates efforts to pinpoint the precise contributions to phenotypic traits. This review aims to investigate the ecological dynamics of microbiomes by examining tools to profile and interpret the role of microbial communities in supporting body site functions and influencing phenotypic outcomes. Drawing from extensive human and germ-free animal microbiome studies, we will discuss the microbiome’s role in maintaining organ homeostasis and its function in host disease protection. We will also review current methodologies for microbiome analysis and discuss the data available on the reproductive microbiome, particularly the bovine vaginal microbiome, and its implications for fertility.

Historically, host-microorganism interactions were viewed primarily through a lens of pathogenicity, emphasizing microorganisms’ role in disease. However, for decades, techniques used in microbiology only allowed researchers to identify and study a small proportion of bacteria due to ordinary bacteriologic techniques which relied on cultures of bacteria in selective medias and oxygen presence, and thus neglecting the majority of bacterial communities that cannot be cultured under these conditions (Dubos et al., 1967). As microbiology evolved, particularly through studies of the gastrointestinal microbiota, researchers recognized that bacteria do not inherently correlate with disease. This discovery shifted perspectives toward understanding certain bacterial relationships as commensal, where bacteria benefit from host resources without causing negative consequences to the host (Young, 2017). Currently, with the advancement of sequence-based technologies, all microorganisms in an environment can be identified without relying on microbial cultures. These techniques amplify DNA sequences, allowing for the identification of an entire microbial community, thereby providing a more comprehensive analysis of the microbiome. Today, it is understood that most host-microbe relationships are symbiotic, where microorganisms play vital roles in the physiology of the body site they inhabit, and disruptions to this balanced ecosystem can enable opportunistic pathogens to proliferate and cause disease (Young, 2017).

The microbiome, encompassing both biotic and abiotic factors, constitutes a complex micro-environment. Biotic factors include not only the diverse microorganisms that inhabit a body site and their metabolic contributions, but also the influence of host genetics, immune responses, and metabolic processes. Abiotic factors, such as temperature, pH, and oxygen availability, also significantly impact the environment, determining which microorganisms thrive in those circumstances (Garcia-Velasco et al., 2017). Thus, microbiome studies aim to characterize the microbial community structure (diversity of species in a healthy state) and function (microbial contributions to physiological processes). Research also investigates how microbial community disturbances (perturbations) correlate with disease or impaired organ function, and how microbial structure and function may change post-disturbance (Katharine and Coyte, 2015). Furthermore, an essential area of microbiome research examines how disturbances shape microbial community resilience, described as the ability of the microbiome to revert to an original state following a perturbation, and explores interventions, such as the use of probiotics, to restore or maintain a balanced microbiome (Suez et al., 2018).

In scientific literature, microbiomes are often considered ecosystems with distinct ecological dynamics. We explored two ecological hypotheses to interpret the importance of microbial communities within a body site, the Walker’s “drivers and passengers” hypothesis (Walker, 1995) and the “Rivet” hypothesis (Garry Peterson and Holling, 1998), both of which offer valuable perspectives on the interactions and roles of microbes within these ecosystems.

### Understanding microbiome structure and function through Walker’s “drivers and passengers” and the “rivet” hypotheses of ecology

An ecosystem is defined as a community of organisms and the network of interactions among them, which together produce a specific function. The function of an ecosystem is often assessed based on a key variable, for example, the conservation of biodiversity or the maintenance of certain environmental conditions over time, such as oxygen production (Mageau, 1999). Walker’s “drivers and passengers” hypothesis (Walker, 1995) suggests that species in an ecosystem can be categorized according to the ecological roles they play. According to Walker, a select group of species, termed “driver” species, are critical to the ecosystem’s function, while other species, termed “true passengers,” contribute minimally to ecosystem dynamics or function. Thus, environmental disturbances that impact “true passenger” communities have little effect on the ecosystem’s function, whereas disturbances affecting “driver” species can disrupt ecosystem function significantly and prove detrimental to the ecosystem. Walker also introduces a third group of species that may initially serve a “passenger-like” role but could become “drivers” if a disturbance changes the ecosystem dynamics. Walker illustrates this with an example: if an environment loses its only nectar-producing species, all nectar-dependent species would disappear, and, subsequently, plants relying on those species for pollination would also be affected. In this scenario, the only nectar-producing species is considered the ecosystem’s “driver”.

The “Rivet” hypothesis (Garry Peterson and Holling, 1998) proposes a slightly different perspective, suggesting that many species in an ecosystem have overlapping functions. Since the functional contributions of a single species are limited, an increase in species diversity promotes both functional diversity and ecosystem stability. The overlapping functions provided by different species implies that losing one species does not immediately result in functional loss. The analogy that encompasses this hypothesis compares ecosystem function to the rivets that secure an airplane wing. While each rivet is essential to maintain the function of the wing, multiple rivets can be lost before the wing’s integrity is compromised, as the remaining rivets continue to support the same function.

While these two hypotheses are not mutually exclusive, they emphasize different aspects of ecosystem importance. The “Rivet” hypothesis suggests that conservation efforts should focus on maintaining species diversity, whereas Walker’s hypothesis advocates for protecting “driver” species to preserve ecological functions. Regardless of which hypothesis better explains microbial roles in specific body environments, studies on microbiome dynamics rely on technologies that identify the microbial community structure and examine the functional contributions of the microbiota within the system.

### Technologies for inferring microbiome structure and function

The primary and most popular method used to investigate microbiome structure is based on amplifying DNA sequences that encode the 16S ribosomal RNA (rRNA) gene. This technique involves extracting DNA from a sample, amplifying the DNA using polymerase chain reaction (PCR), and performing high-throughput sequencing. The 16S rRNA gene is used because it is universally present across microorganisms and contains both conserved and variable regions. These characteristics make it an ideal target, allowing researchers to group sequences into different operational taxonomic units (OTUs) according to sequence similarities and differences. The amplified gene sequences can then be cross-referenced with genome libraries, enabling taxonomic classification attributed to known phyla, genera, or even species levels. Data generated from 16S rRNA analysis provides insights into relative abundances of OTUs, associations between OTUs and phenotypes, OTU diversity within an environment, and phylogenetic relationships (Janda and Abbott, 2007). However, while 16S rRNA analysis is highly effective for identifying microbiome structure, it only indirectly infers function. For example, if the analysis identifies *Escherichia coli*, it does not distinguish between strains, which may have vastly different roles. Some *E. coli* strains are beneficial, promoting symbiosis with the host, while others, like *E. coli* O157, are highly pathogenic (Huang et al., 2014).

To investigate microbiome function more directly, a more comprehensive approach is required, involving whole-genome shotgun (WGS) sequencing. This metagenomic approach sequences the entire community genome by fragmenting all DNA from a sample and analyzing it through high-throughput sequencing. Unlike 16S rRNA, which targets a single gene, WGS captures all genomic data in the community. Cross-referencing the generated sequences with genome libraries enables researchers to determine community structure, identify specific genes, and reconstruct metabolic pathways, allowing for a functional assessment of the microbiome (Chen and Pachter, 2005). However, it is important to note that both 16S rRNA and WGS analyze DNA from the microbiome, regardless of whether the DNA originated from living or dead microbes, thus results must be interpreted with caution.

Metatranscriptomics offers another approach for inferring microbiome function by targeting RNA. In this method, expressed RNA from a sample is reverse transcribed into DNA, sequenced, and analyzed, to reveal genes that are actively transcribed in the environment. Other functional assessment methods focus on detecting proteins (proteomics) or metabolites (metabolomics) present in the environment through mass spectrometry, which allows researchers to quantify these components (Huang et al., 2014). Although metabolomics and proteomics are valuable for evaluating microbiome function, they also capture metabolites and proteins from the host, thus results also require careful interpretation.

### The relevance of the microbiome in homeostasis and disease

Microbiota, referring to the microorganisms within a given microbiome, play an essential role in homeostasis by interacting closely with the host and organ environment. Often, microbiota introduce functions unique to microbes, providing benefits otherwise absent in a sterile environment (Young, 2017). Additionally, the microbiota may augment pathways encoded by the host, enhancing organ function. Through metabolic by-products, these microbes can also create environmental conditions unfavorable to pathogenic bacteria, helping to suppress disease (Greenbaum et al., 2019). In this way, the microbiome contributes by (1) supporting the homeostatic function of organs and (2) establishing an environment that limits the growth of pathogenic organisms, and thus protects the host from disease.

### The microbiota’s role in organ homeostasis

Insights from germ-free animal models are valuable to illustrate the role of microbiota in maintaining homeostasis of an environment. While many animal species can survive in sterile conditions, indicating the microbe-host relationship is not necessary for life, this germ-free state often results in anatomical and physiological deficiencies. For example, germ-free mice exhibit an enlarged cecum, making up 20–25% of body weight, with an intestinal mucosa presentation that resembles the pre-natal-like state (Dubos et al., 1967). This cecal enlargement is the result of the buildup of dietary fiber that, under normal conditions, would be fermented by the natural intestinal microbiota (Furusawa et al., 2013). Germ-free animals also present compromised immune systems. In germ-free guinea pigs, there is decreased immune cell accumulation in the intestinal lining and abnormal intestinal histology (Dubos et al., 1967). Moreover, bacterial-derived metabolites, such as the short-chain fatty acid butyrate, play a crucial role in inducing regulatory T-cell function in the intestines (Furusawa et al., 2013). Importantly, many of these structural and functional impairments in germ-free animals can be restored through the introduction of proper bacterial species.

Microbiota is critical for proper gastrointestinal function, which is well-studied in mammals. The human gut, for example, hosts trillions of bacteria (Fuloria et al., 2022) and although microbial genomes are smaller than the host’s, the microbes collectively perform extensive metabolic functions (Young, 2017). For example, when microbiota from conventionally raised mice is introduced to germ-free mice, body fat increases within weeks despite decreased food intake, demonstrating the microbiota’s role in energy and nutrient harvesting from the diet (Backhed et al., 2004). Certain bacteria, however, can digest compounds like phosphatidylcholine in feed to produce trimethylamine, a precursor to trimethylamine-N-oxide, which induces metabolic changes linked to cardiovascular disease (Wang et al., 2011). Thus, microbiota metabolism in the gut significantly influences the types of molecules available for host absorption into the systemic circulation, and significantly contributes to host metabolism (Dubos et al., 1967).

One of the most comprehensive microbiome studies to date, the Human Microbiome Project Consortium (2012), examined microbiome structure and function across various human body sites in healthy individuals using both 16S rRNA and metagenomics techniques. Findings revealed that each body site’s microbiota composition differs significantly among healthy individuals, indicating no universal core microbiome structure associated with health. In fact, factors such as ethnicity, geography, and lifestyle (e.g., diet) have been identified to shape microbiome structure (Arumugam et al., 2011). Despite structural variation, the Human Microbiome Project found that metabolic pathways expressed in the microbiomes of different healthy individuals were well conserved, suggesting that while a universal core composition may not exist, a core microbiome function does correlate with health. This aligns with the “Rivet” hypothesis, in which different “healthy” microbiomes consist of diverse microbial communities in the same way that different airplanes may have their wings secured by different kinds of rivets. What must be conserved in healthy environments, however, is the function of the microbiome that relates to health, in the same way that a functional airplane is one in which the wings are always secured during flight. However, perturbations in the microbiome can disrupt this balance, often leading to dysbiosis and improper homeostasis of a body site that may evolve to disease.

Microbiome disruptions, whether from antibiotics, dietary changes, or other sources, can lead to changes in microbiome composition and function associated with disease (Schmidt et al., 2018). The obesity model is valuable for studying these relationships within the microbiota. Obese individuals, for instance, tend to have a greater abundance of the phylum Firmicutes and lesser levels of Bacteroidetes compared to healthy individuals. As obese individuals lose weight through hypocaloric diets, this microbial imbalance stabilizes, suggesting that the obese phenotype corresponds with altered gut microbiome structure (Turnbaugh et al., 2006). Functional changes in the microbiome also accompany obesity. In leptin-knockout mice models, the obese microbiome demonstrates an enhanced ability to extract energy from food, evident from reduced energy availability in feces along with WGS that reveals enriched pathways involved with glucose metabolism, enzymes capable of breaking down otherwise host-indigestible carbohydrates, and enzymes involved in fermentation products (Turnbaugh et al., 2006). When germ-free mice receive microbiota from either obese mice or lean littermates, those receiving the obese microbiome exhibit greater Firmicutes abundance, Bacteroidetes abundance reduction, and increased body fat deposition, underscoring the microbiome’s influence on the host’s energy balance. From an ecological perspective, a diet rich in calories favors the proliferation of Firmicutes, enhancing the microbiome’s capacity to break down nutrients in an energy-dense environment. This in turn makes more energy available for host absorption, promoting obesity. Conversely, as dietary calories decrease, less energy becomes available in the gut environment, creating an environment less favorable for Firmicutes and supporting reestablishment of Bacteroidetes. In this scenario, dietary energy availability acts as a perturbation factor, selectively altering the microbiome’s structure and function leading to disruptions in gut homeostasis to ultimately favor obesity.

### The importance of the microbiome in controlling pathogenic microorganisms: insights from the human vaginal microbiome

A key role of the microbiota-host symbiosis is the ability of microbiota to inhibit the proliferation of pathogenic microorganisms (Fuloria et al., 2022). When this symbiotic balance is disrupted, disease often follows. While the gastrointestinal tract hosts a wide diversity of species in its nutrient-rich environment, the human vaginal microbiome is generally less diverse and typically dominated by *Lactobacillus* species (Garcia-Velasco et al., 2017). Lactobacilli produce lactic acid as part of their metabolism, promoting an acidic environment which is favorable to their own growth but hostile to many other microbes (Garcia-Velasco et al., 2020). This acidic environment contributes to the vagina’s immune defenses, helping protect the upper reproductive and urinary tracts from pathogenic bacteria (Garcia-Velasco et al., 2020). When *Lactobacillus* species are depleted, a condition known as bacterial vaginosis (BV) can develop, characterized by greater bacterial diversity, increased vaginal pH, and anaerobic bacterial growth (Lamont et al., 2011). Bacterial vaginosis often leads to vaginal discharge and malodor and is linked to complications such as preterm birth, pregnancy loss, infertility, endometritis, and increased vulnerability to HIV and other sexually transmitted infections (Taha et al., 1998; Hickey et al., 2012).

Five microbial community state types (CSTs) have been identified in the human vaginal microbiome and are considered to compose the natural, healthy vaginal microbiome. Four CSTs (I, II, III, and V) are dominated by specific *Lactobacillus* species (*L. iners, L. crispatus, L. jensenii, and L. gasseri*). The CST-IV is unique in its composition as it is composed mainly of anaerobic bacteria including *Gardnerella* and *Ureaplasma* (Garcia-Velasco et al., 2020). Host genetics seem to influence CST type, as CST-IV is more common in women of Hispanic and African-American descent, while the other CSTs are more prevalent in European and Asian women (Ravel et al., 2011). Although CST-IV is found in healthy women, African-American women have a greater prevalence of BV than white non-Hispanic women, suggesting that some CST compositions may be more susceptible to dysbiosis and BV (Smith and Ravel, 2017). Furthermore, throughout a woman’s life, factors such as menstrual cycle stage, pregnancy, sexual activity, antibiotic use, smoking, and personal hygiene can influence the vaginal microbiome (Garcia-Velasco et al., 2020).

Hormonal fluctuations, especially during the menstrual cycle, play a major role in maintaining a healthy vaginal microbiome. Estrogens are known to promote epithelial cell growth and glycogen storage in vaginal epithelial cells (Cribby et al., 2008), while progesterone (P4) induces epithelial cytolysis, releasing glycogen that bacteria can then use as an energy source (Garcia-Velasco et al., 2017). Greater glycogen levels favor glucose-fermenting bacteria like *Lactobacillus*, helping maintain an acidic vaginal environment. By contrast, prepubertal girls have a neutral to slightly alkaline vaginal environment, with microbiota similar to that found on skin and in the gut (Hickey et al., 2012). Opposingly, postmenopausal women experience reduced estrogen production, leading to lower vaginal glycogen availability and a decline in *Lactobacillus* abundance, increasing their risk of BV (Cauci et al., 2002). Hormone replacement therapy can help by restoring estrogen levels, which increases glycogen availability and supports *Lactobacillus* dominance, promoting an acidic vaginal pH not typically observed in postmenopausal women who do not undergo hormone therapy (Huang et al., 2014).

*Lactobacillus* dominance is particularly important for fertility and pregnancy outcomes (Xu et al., 2020). Women undergoing *in vitro* fertilization (IVF) who have lower *Lactobacillus* abundance are less likely to conceive successfully (Koedooder et al., 2019). In fact, classifying women based on *Lactobacillus* abundance have been found to predict IVF success rates, with only 5.9% of women in the low-*Lactobacillus* group achieving pregnancy, compared to a 52% success rate in the high-*Lactobacillus* group (Koedooder et al., 2019). Beyond fertility, a *Lactobacillus*-rich vaginal microbiome is crucial for sustaining pregnancy. As gestation progresses, the composition of the vaginal microbiome changes, characterized by a loss in bacterial diversity and predominance of *Lactobacillus* species (Aagaard et al., 2012). Disruptions in this vaginal microbiome profile have been linked to preterm birth (Garcia-Velasco et al., 2020), where only about 3% of women who carry to term have an altered vaginal microbiome. Additionally, a reduction in *Lactobacillus* and increased microbial diversity are often observed before membrane rupture, a major cause of preterm birth (Brown et al., 2019).

The human vaginal microbiome aligns with the ecological “driver and passenger” hypothesis, where *Lactobacillus* species “drive” the environment by maintaining an acidic pH that limits the establishment and growth of pathogenic bacteria, thus protecting the host from disease. Interestingly, humans are unique among mammals in having an acidic vaginal microbiome. In a study of the vaginal microbiomes of 22 mammalian species, only humans were found to have a vaginal pH lower than 4.5 in healthy conditions (Miller et al., 2016). In fact, while BV shifts vaginal pH to values between 5 and 5.5, the lowest pH in healthy non-human mammals was above 6. Although *Lactobacillus* species were found in most of these non-human mammal species, their relative abundance was typically below 2% (Miller et al., 2016). These findings underscore the uniqueness of the human vaginal microbiome in maintaining a highly acidic environment.

### The bovine reproductive microbiome

The main features and findings related to the bovine and human vaginal microbiome are summarized in Table 1. The bovine reproductive microbiome differs significantly from the human vaginal microbiome, both in structure and implications for reproductive health. Unlike the human vaginal microbiome, which is dominated by *Lactobacillus* species that produce lactic acid, maintaining an acidic pH to inhibit pathogenic growth, the bovine vaginal environment lacks a similar *Lactobacillus*-dominant structure and is rather a diverse environment, characterized by a near-neutral pH (Clemmons et al., 2017; Swartz et al., 2014), and composed mainly by Bacteria, alongside little abundance of Archea and Eukaryote microorganisms (Laguardia-Nascimento et al., 2015). Studies using 16S rRNA sequencing have consistently reported that Firmicutes is the most abundant phylum in the bovine vagina, with relative abundances ranging widely across studies from as little as 31.5% to as great as 64% (Deng et al., 2019; Chen et al., 2020; Miranda-Caso Luengo et al., 2019). Although bacteria from the genus *Lactobacillus* belong to the phylum Firmicutes, studies report no relevant abundance of *Lactobacillus* in the bovine vaginal flora (Swartz et al., 2014). The second most abundant bacteria found in the bovine reproductive microbiome belong to the phyla *Bacteroidetes* and Proteobacteria (Clemmons et al., 2017; Laguardia-Nascimento et al., 2015). Interestingly, other studies have identified Tenericutes and Fusobacteria as significant components of the vaginal microbiome of beef heifers synchronized for estrus and subjected to timed-artificial insemination (Messman et al., 2020), which suggests that factors such as age, diet, location, hormonal concentrations, and sexual maturity might impact the microbial composition, though these associations remain speculative.

**Table 1:** Summary of features and main findings of the human and bovine vaginal microbiomes.

Feature	Bovine vaginal microbiome	References	Human Vaginal Microbiome	References
Phyla	Most abundant: Firmicutes^1-7^; Tenericutes^8^ 2nd most abundant: Proteobacteria^4,6,8^ Bacteroidetes^2,3,5^	Laguardia-Nascimento et al. (2015)^1^, Clemmons et al. (2017)^2^, Moore et al. (2017)^3^, Deng^4^, Miranda-Caso Luengo et al. (2019)^5^, Chen and Pachter (2005)^6^, Quadros et al. (2020)^7^, and Messman et al. (2020)^8^	Most abundant: Firmicutes^1-3^; Others: Actinomycetes^2^ Actinobacteria^3^ Bacteroidetes^3^ Proteobacteria^3^ Fusobacteria^3^ Tenericutes^3^	Miller et al. (2016)^1^, Lamont et al. (2011)^2^, and Xu et al. (2020)^3^
Most enriched genera or species (relative abundance)	Bacteroides, Streptococcus, Campylobacter^1^; Aeribacillus, Bacteroides, Clostridium, Ruminococcus, Rikenella, Alistipes, Bacillus, Eubacterium, Prevotella, non-classified bacteria^2^; undetermined genus (3.4%), genus 5-7 N15 (3.4%), Oscillospira (2.0%), Butyrivibrio (1.8%), Ureaplasma (1.8%), Campylobacter (1.7%), Dorea (1.6%), Paraprevotellaceae (1.5%), Clostridium (1.3%), Helococcus (1.2%), Corynebacterium (1.0%)^3^; unclassified Enterobacteriaceae (21.05%), Ureaplasma (4.37%), unclassified Bacteroidaceae (2.49%)^4^	Miranda-Caso Luengo et al. (2019)^1^, Laguardia-Nascimento et al. (2015)^2^, Clemmons et al. (2017)^3^, and Deng et al. (2019)^4^	*Lactobacillus^1-5^: L. iners, L. cripsatus, L. jensenii, L. gasseri* *Pseudomonas* ^2^ *Streptococcus* ^2^ *Gardnerella vaginalis* ^1-6^ *Ureaplasma* ^1,2,6^ *Prevotella* ^6^ *Corynebacterium* ^6^ *Atopobium* ^6^ *Megasphaera* ^6^ *Sneathia* ^6^	Miller et al. (2016)^1^, Lamont et al. (2011)^2^, Xu et al. (2020)^3^, Garcia-Velasco et al. (2017)^4^, Garcia-Velasco et al. (2020)^5^, and Ravel et al. (2011)^6^
Mean pH	6.15–7.44^1^; 7.3^2^	Clemmons et al. (2017)^1^ and Swartz et al. (2014)^2^	Healthy: < 4.5^1^; > 4.5^1^ Bacterial Vaginosis: 5–5.5^2^; > 4.5^3^	Miller et al. (2016)^1^, Lamont et al. (2011)^2^, and Huang et al. (2014)^3^
Main findings	Decreased diversity in pregnant animals, and profile of OTUs based on aerotolerance^1^; Abundance of OTUs based on estradiol concentrations^2^; Greater diversity in vagina prior to ES in cows that became pregnant^3^. Resident microbiome of the virgin and pregnant uteri^4^; greater diversity and microbial richness in the vagina compared to the uterus^5^; dysbiotic microbiome 7 days postpartum in cows that developed endometritis^6^	Laguardia-Nascimento et al. (2015)^1^, Messman et al. (2020)^2^, Ault et al. (2019)^3^, Moore et al. (2017)^4^, Clemmons et al. (2017)^5^, and Miranda-Caso Luengo et al. (2019)^6^	Dominated by Lactobacillus species that are categorized into community states^1-5^ Decreased Lactobacillus can result in bacterial vaginosis and other reproductive-related issues^2-3,8–9^ Women of Hispanic and African-American ethnicities tend to have lower Lactobacillus prevalence^6^ Great estrogen aids in Lactobacillus dominance during the estrous cycle^1,7,10–11^	Miller et al. (2016)^1^, Lamont et al. (2011)^2^, Xu et al. (2020)^3^, Garcia-Velasco et al. (2017)^4^, Garcia-Velasco et al. (2020)^5^, Huang et al. (2014)^7^, Taha et al. (1998)^8^, Hickey et al. (2012)^9^, Cribby et al. (2008)^10^, and Cauci et al. (2002)^11^

^1-11  Superscripts correspond to the references cited for each statement or finding.^

### Hormonal contributions to the bovine vaginal microbiome

While the role of reproductive steroid hormones in maintaining the healthy human vaginal microbiome is well established, the role of steroid reproductive hormones in modulating the bovine vaginal microbiome is not fully understood. In a study aimed at investigating the role of P4 in vaginal microbiome composition, Quadros and colleagues reported an increased relative abundance of members from Family XIII AD3011 and Family XIII unclassified in vaginal samples of Holstein cows one day after the removal of a controlled internal drug release (CIDR) device containing P4, compared to samples taken immediately before CIDR insertion (Quadros et al., 2020). Since these bacteria are often found in ruminal samples (Petri et al., 2020), it remains unclear whether the observed differences in bacterial abundance after CIDR use are driven by varying concentrations of P4 or by the potential acquisition of these bacteria from bovine feces, with the CIDR possibly serving as a vector. In fact, greater diversity and microbial richness have been observed in the vagina of cows compared to the uterus (Clemmons et al., 2017), and researchers attribute this difference to the vagina’s proximity to the external environment, as many microorganisms found in the vagina in this study are also commonly found in the gastrointestinal tract. Recently, we reported that the use of a CIDR during an estrus synchronization protocol in cows resulted in vaginal inflammation, an increase in vaginal pH, and a decreased diversity of the microbiota, characterized by reduced abundance of anaerobic bacteria (Wege Dias, 2023). However, we cannot conclude whether the changes in the vaginal microbiome observed in response to the CIDR are due to P4 supplementation, the vaginitis induced by its use, or the device’s role as a vector for microorganisms.

In the study from Laguardia-Nascimento and colleagues, authors explored the vaginal microbiome of pregnant and non-pregnant Nellore cows and heifers and reported decreased diversity in the vaginal microbiome during the first trimester of gestation when compared to non-pregnant animals. Although neither hormone concentrations nor ovarian structures were measured to determine the influence of steroid hormones on the microbiome’s community composition, it can be presumed that the vagina of pregnant animals had a longer exposure to elevated P4 concentrations, which is characteristic of pregnancy, compared to non-pregnant animals. This finding, in turn, could be an important factor in selecting the vaginal microorganisms that thrive and decrease bacterial diversity, as observed during gestation in humans. Furthermore, Deng and colleagues investigated the vaginal microbiome of heifers from the onset of the breeding season throughout pregnancy and reported fewer OTUs observed in the vagina of pregnant heifers in the third trimester compared to the second trimester of gestation. However, both pregnant and non-pregnant animals showed an increase in bacterial diversity in the vagina from the pre-breeding period to the second trimester of gestation (Deng et al., 2019). Collectively, while the available data suggest P4 plays a role in modulating the bovine vaginal microbiome, the exact role as to which P4 selects microorganisms in the bovine vagina remains unclear.

Regarding the role of estrogens in the composition of the bovine vaginal microbiome, classification of the vaginal microbiome of heifers at timed artificial insemination (TAI) showed no effect of estradiol concentrations on microbiome diversity (Messman et al., 2020). The authors, however, reported a differential abundance of seven OTUs based on estradiol classification, with a greater abundance of *Leptotrichiaceae* unclassified, *P. multocida*, and *Pasteurellaceae* unclassified, and a lower abundance of *Histophilus somni*, *Actinobacillus seminist*, *Bacteroidetes* unclassified, and *Fusobacterium* unclassified in the vaginas of heifers classified as high estradiol compared to heifers classified as either medium or low estradiol. This study, however, is the only one to report a vaginal microbiome not dominated by bacteria from the phylum Firmicutes, but rather by Tenericutes. In addition, the relevance of these bacteria for reproduction and fertility remains unclear. Nevertheless, this study demonstrates an effect of estradiol on the relative abundance of bacteria in the vagina. Whether this is due to a direct effect of estradiol or to abiotic changes in the environment, such as vaginal pH as seen in women, or to the greater amount of cervical mucus production, as characteristic of estrus, is also unclear. Longitudinal studies investigating shifts in vaginal microbial composition before and after puberty attainment would be valuable to elucidate the effects of steroid hormones on the vaginal microbiome and are currently lacking in literature.

### Features of the bovine vaginal microbiome related to fertility

Identification of features of the bovine vaginal microbiome that relate to fertility are valuable for the bovine industry and would allow for the development of interventions that lead to greater fertility; however, no single core structure of the bovine vaginal microbiome composition has been identified. One study found greater abundance of *Histophilus somni*, *Clostridiaceae 02d06*, and *Campylobacter* in the vaginas of heifers that failed to become pregnant from samples taken at the onset of the breeding season (Deng et al., 2019). In another study, Holstein cows were pre-synchronized with an injection of prostaglandin F-2 alpha and inseminated based on estrus expression (Chen et al., 2020). Cows that failed to conceive were observed for estrus and assigned to be artificially inseminated up to three times. Vaginal microbiome samples were collected at each of the three AI events, and researchers found no differences in bacterial diversity in relation to fertility outcomes. Nevertheless, they reported five OTUs enriched in cows that became pregnant and 21 OTUs enriched in cows that did not at the first AI service. In addition, at the second AI, seven OTUs were more abundant in cows that became pregnant, and six OTUs were more abundant in cows that did not. While these results suggest a possible effect of the enriched OTUs on fertility outcomes, none of the enriched OTUs overlapped between the two AI occasions (Chen et al., 2020). Although some of these findings indicate a difference in the abundance of specific OTUs related to fertility outcomes, the function of these OTUs within the vaginal environment is often unknown and speculative, making it difficult to understand the actual contributions of these OTU abundances to fertility. Furthermore, these studies did not investigate other parameters related to microbiome function, such as pH, oxygen availability, or enriched metabolic pathways, which could help explain functional features of the microbiome that relate to fertility outcomes.

While no core composition of the bovine vaginal microbiome has been identified to relate to fertility, it seems that greater diversity in the bovine vaginal microbiome is beneficial to fertility. In one study, researchers investigated the vaginal and uterine microbiome of cows enrolled in an estrous synchronization (ES) protocol not involving the use of a CIDR and reported greater microbiome diversity in the vaginas of cows that conceived to the protocol 21 days prior to TAI, but not at protocol initiation (9 days prior to TAI), nor 2 days prior to TAI (Ault et al., 2019). Accordingly, we have recently reported similar results, where cows that became pregnant to TAI following a CIDR-based ES had greater vaginal microbiome diversity in samples collected immediately prior to CIDR insertion when compared to cows that did not conceive to the protocol (Wege Dias, 2023). Furthermore, we found that cows that conceived to the protocol had a narrow, near-neutral distribution of vaginal pH at TAI, while cows that failed to conceive to the protocol had a wide range of vaginal pH distributions at TAI.

In the study by Laguardia-Nascimento et al. (2015), researchers did not find differences in the vaginal microbiome composition between cows and heifers, nor between pregnant and non-pregnant animals. However, when analyzing the abundance of the ten most enriched OTUs found in individual samples, researchers identified that animals could be classified into three groups based on the aerotolerance of bacteria at the genus level. Animals classified as “Group A” had a greater abundance of aerobic and facultative anaerobic OTUs, those in “Group B” showed an intermediate abundance of aerotolerant OTUs, and animals in “Group C” were found to have only obligate anaerobic OTUs present. The researchers attributed these differences to possible anatomical variations in the vagina that might allow for air to accumulate, thereby selecting for these OTUs. Although no specific group in this study was associated with pregnancy status, as both pregnant and non-pregnant animals were identified in each group, classifying microbiomes based on functional characteristics is essential for determining the functional microbiome features of the bovine vagina that might relate to fertility, pregnancy development, and protection against pathogens.

### The bovine uterine microbiome and its relationships to fertility and health

Previous research upheld the belief that a sterile uterus was necessary to establish and maintain pregnancy. It was thought that events such as parturition or artificial insemination acted as catalysts allowing bacteria to colonize the uterus and cause disease. However, recent studies into the reproductive microbiome have challenged this perspective. In a recent study, the 16S rRNA approach was used to investigate microbiome composition in samples collected aseptically from virgin and pregnant uteri. Researchers reported a resident microbiome composed mainly of Firmicutes, Bacteroidetes, and Proteobacteria in both virgin and pregnant uteri (Moore et al., 2017).

In another study investigating the uterine microbiome of postpartum beef cows one and three days prior to TAI, researchers reported a similar overall microbiome composition between cows that conceived to TAI and those that did not. Despite a similar overall composition, they observed an elevated abundance of *Blautia*, *Butyrivibrio*, and *Natronincola* genera, along with increased concentrations of pro-inflammatory cytokines in uterine samples of females unable to establish pregnancy (Smith et al., 2023).

Regarding endometrial health, a study investigated the uterine and vaginal microbiomes of dairy cows prior to calving, as well as at 7 days postpartum (DPP), 21 DPP, and 50 DPP. Researchers found that a dysbiotic vaginal microbiome at 7 DPP, characterized by a decreased presence of OTUs highly abundant in healthy cows, along with the appearance of a sub-community associated with endometritis, correlated with a greater prevalence of endometritis at 21 DPP (Miranda-Caso Luengo et al., 2019). Furthermore, they reported dynamic temporal changes to the reproductive microbiome. Soon after calving, the vaginal and uterine microbiomes were similar in OTU composition and abundance. As the postpartum period progressed, however, these similarities decreased, and by 50 DPP, the reproductive microbiome of healthy cows resembled the pre-calving microbiome. Additionally, similarities between uterine and vaginal microbiomes were greater at 7 DPP in cows that developed endometritis compared to healthy cows assessed at 21 DPP. The vaginal microbiome at 7 DPP related to the development of endometritis and was characterized by a decreased abundance of observed OTUs and decreased microbial diversity. These changes included a decrease in the abundance of bacteria from the phylum Firmicutes and an associated increase in bacteria from the phylum Bacteroidetes, with cows that developed endometritis exhibiting a decreased Firmicutes to Bacteroidetes ratio and a greater dominance of OTUs from the *Streptococcus* genus.

Collectively, the conservation of the human vaginal microbiome likely falls under the “Walker’s” hypothesis, in which maintaining the *Lactobacillus* “driver” species in the environment is important to maintain an acidic vaginal pH related to health. In cattle on the other hand, conservation of vaginal health seems to fall under the “Rivet’s” hypothesis, in which a healthy reproductive microbiome is associated with vaginal bacterial diversity, as observed in cows that conceived following an ES protocol in the studies from Ault et al. (2019) and Wege Dias (2023). In turn, perturbations in this environment, as caused by parturition, results in an environment that allows for bacteria to thrive and cause disease, as in the case of bacteria from the genus *Streptococcus* in the study from Miranda-Caso Luengo et al. (2019).

In terms of fertility outcomes, the bovine vaginal microbiome does not have a single core composition predictive of successful conception, contrasting with humans where *Lactobacillus* dominance correlates with higher fertility and pregnancy maintenance. Instead, studies have identified various OTUs linked to successful pregnancies, but these differ by sampling period and reproductive phase, with no OTUs consistently predictive across all studies (Chen et al., 2020; Ault et al., 2019).

An essential aspect in cattle is the relationship between microbiome diversity and reproductive health, particularly postpartum. The postpartum period, especially shortly after calving, is marked by significant changes in both the vaginal and uterine microbiomes, with reduced diversity and specific microbial shifts associated with endometritis development. Notably, cows with a dysbiotic vaginal microbiome early postpartum were more likely to develop endometritis, marked by an increase in *Streptococcus* and a shift in the Firmicutes-to-Bacteroidetes ratio, indicating a propensity for pathogenic colonization when diversity is disrupted (Miranda-Caso Luengo et al., 2019).

### Potential strategies for intervention in the vaginal microbiome

Studies aimed at understanding the function of the vaginal microbiome are essential for defining a functionally healthy vaginal ecosystem and for developing potential intervention strategies. While the relative abundance of *Lactobacillus* in the bovine vagina is very low (Swartz et al., 2014), the role of *Lactobacillus* species in maintaining an acidic vaginal pH and protecting against pathogens is well-established in humans. In bovines, however, the vaginal microbiota appears to be heavily influenced by gastrointestinal microbiota, likely due to the proximity of the anus to the vulva, which allows for constant exposure to fecal microbiota (Laguardia-Nascimento et al., 2015). It remains unclear, however, whether the bovine vagina is inherently unsuitable for *Lactobacillus* growth, or if constant introduction of bacteria from feces enables gastrointestinal bacteria to thrive in place of *Lactobacillus* species.

Administering probiotics that include lactic acid-producing bacterial strains (*Lactobacillus rhamnosus*, *Lactobacillus reuteri*, and *Pediococcus acidilactici*) intravaginally, twice a week for three weeks prior to calving, has been shown to decrease postpartum endometritis in dairy cows (Genis et al., 2018). The treated cows exhibited decreased phagocytosis activity of polymorphonuclear (PMN) leukocytes, as analyzed by PMN gene expression, indicating a reduced uterine challenge from pathogenic bacteria in this group. In a study involving ewes, administration of lactic acid-producing bacteria concurrently with intravaginal sponges used for P4 delivery led to decreased abundance of PMN leukocytes two days after sponge removal (Quereda et al., 2020). In this study, fertility rates were 91% for probiotic-treated ewes compared to 60% for controls, although this difference was not statistically significant due to the small sample size. In both studies, however, no metagenomic approach was employed to assess structural changes in the vaginal microbiome in response to probiotic administration, nor was vaginal pH measured. Nevertheless, the beneficial outcomes observed support further investigation into the ecological responses of the bovine vaginal microbiome to lactic acid-producing bacteria, which may reveal potential health and fertility benefits.

In humans, antibiotic usage frequently disrupts microbial communities, resulting in dysbiosis within the gastrointestinal tract. This dysbiosis can have both metabolic and pathological consequences. Efforts to restore the intestinal microbiota in humans following antibiotic treatment have included probiotics and autologous fecal matter transfer (aFMT). A recent study investigated the ability of probiotics versus aFMT in restoring the original microbial composition in both human and mouse subjects (Suez et al., 2018). Participants received either commercially available probiotics, their own pre-antibiotic microbiome through aFMT, or no intervention to allow spontaneous recovery after antibiotics. Notably, probiotics were found to inhibit the intestinal tract’s capacity to return to its natural state. In mice, aFMT and spontaneous recovery groups regained bacterial richness within eight days, while the probiotic-treated group failed to reach similar diversity levels even four weeks post-treatment. Additionally, four bacterial taxa in the probiotics were inversely correlated with stool sample diversity, suggesting that these species were able to establish in the gut and impede microbiome reconstitution. In humans, antibiotic treatment significantly reduced microbial diversity, and probiotics led to seven of the eleven species in the supplement remaining elevated for up to five months post-treatment. By contrast, the aFMT and spontaneous recovery groups returned to baseline diversity within one and twenty-one days, respectively. Moreover, multiple metabolic pathways were restored in the aFMT and spontaneous groups but not in the probiotics group, with pathway expression inversely correlated with microbial diversity.

These findings illustrate how antibiotics can severely deplete intestinal microbial communities, allowing probiotic species to dominate and hinder the microbiome’s recovery to its pre-antibiotic state. Restoration of the indigenous healthy microbiome appears to be the most effective strategy for reconstituting microbial communities post-antibiotic treatment. In cattle, limited data on the functional microbiome’s role in health and fertility makes it difficult to determine whether promoting an acidic vaginal environment would be beneficial or if using fertile, healthy cows as microbiome donors could offer advantages.

## Conclusion

The role of microbiota in maintaining homeostasis and protecting the host from disease is remarkable. Although research on the bovine reproductive vaginal microbiome is still in its early stages, most studies have focused on identifying a core structural profile of the bovine vaginal microbiome associated with fertility, primarily through the 16S rRNA approach. While these foundational studies have significantly contributed to our understanding, the diverse composition of phyla and genera in the bovine reproductive microbiome, as shown across various studies, suggests that a single, definitive microbiome profile linked to fertility may not exist. Similar findings were observed in The Human Microbiome Project, which found no universal structural profile among healthy human microbiomes. Instead, core metabolic pathways associated with health were consistently conserved. This suggests that while a core structural profile may be absent in the bovine vaginal microbiome, functional characteristics may still be related to health and fertility outcomes. Collectively, the bovine vaginal microbiome is primarily composed of bacteria from the phylum Firmicutes. While little is known about the effects of reproductive hormones in modulating the bovine vaginal microbiome, a greater diversity of microorganisms suggests a more resilient microbiome in response to hormonal interventions commonly used in reproductive practices, as females with greater vaginal microbiome diversity before such interventions have shown greater fertility.

## References

[ref1] Aagaard K. Riehle K. Ma J. Segata N. Mistretta T. A. Coarfa C.. (2012). A metagenomic approach to characterization of the vaginal microbiome signature in pregnancy. PLoS One 7:e36466. doi: 10.1371/journal.pone.0036466, PMID: 22719832 PMC3374618

[ref2] Arumugam M. Raes J. Pelletier E. Le Paslier D. Yamada T. Mende D. R.. (2011). Enterotypes of the human gut microbiome. Nature 473, 174–180. doi: 10.1038/nature09944, PMID: 21508958 PMC3728647

[ref3] Ault T. B. Clemmons B. A. Reese S. T. Dantas F. G. Franco G. A. Smith T. P.. (2019). Uterine and vaginal bacterial community diversity prior to artificial insemination between pregnant and nonpregnant postpartum cows. J. Anim. Sci. 97, 4298–4304. doi: 10.1093/jas/skz210, PMID: 31250893 PMC6776308

[ref4] Backhed F. Ding H. Wang T. Hooper L. V. Koh G. Y. Nagy A.. (2004). The gut microbiota as an environmental factor that regulates fat storage. Proc. Natl. Acad. Sci. USA 101, 15718–15723. doi: 10.1073/pnas.0407076101, PMID: 15505215 PMC524219

[ref5] Brown R. G. Al-Memar M. Marchesi J. R. Lee Y. S. Smith A. Chan D.. (2019). Establishment of vaginal microbiota composition in early pregnancy and its association with subsequent preterm prelabor rupture of the fetal membranes. Transl. Res. 207, 30–43. doi: 10.1016/j.trsl.2018.12.005, PMID: 30633889 PMC6489901

[ref6] Cauci S. Driussi S. De Santo D. Penacchioni P. Iannicelli T. Lanzafame P.. (2002). Prevalence of bacterial vaginosis and vaginal flora changes in peri-and postmenopausal women. J. Clin. Microbiol. 40, 2147–2152. doi: 10.1128/JCM.40.6.2147-2152.2002, PMID: 12037079 PMC130764

[ref7] Chen S. Y. Deng F. Zhang M. Jia X. Lai S. J. (2020). Characterization of vaginal microbiota associated with pregnancy outcomes of artificial insemination in dairy cows. J. Microbiol. Biotechnol. 30, 804–810. doi: 10.4014/jmb.2002.02010, PMID: 32238772 PMC9728155

[ref8] Chen K. Pachter L. (2005). Bioinformatics for whole-genome shotgun sequencing of microbial communities. PLoS Comput. Biol. 1, 106–112. doi: 10.1371/journal.pcbi.0010024, PMID: 16110337 PMC1185649

[ref9] Clemmons B. A. Reese S. T. Dantas F. G. Franco G. A. Smith T. P. L. Adeyosoye O. I.. (2017). Vaginal and uterine bacterial communities in postpartum lactating cows. Front. Microbiol. 8:1047. doi: 10.3389/fmicb.2017.01047, PMID: 28642755 PMC5463355

[ref10] Cribby S. Taylor M. Reid G. (2008). Vaginal microbiota and the use of probiotics. Interdiscip Perspect Infect Dis 2008:256490. doi: 10.1155/2008/256490, PMID: 19343185 PMC2662373

[ref11] Deng F. McClure M. Rorie R. Wang X. Chai J. Wei X.. (2019). The vaginal and fecal microbiomes are related to pregnancy status in beef heifers. J Anim Sci Biotechnol 10:92. doi: 10.1186/s40104-019-0401-2, PMID: 31857897 PMC6909518

[ref12] Dubos R. J. Savage D. C. Schaedler R. W. (1967). The indigenous flora of the gastrointestinal tract. Dis. *Colon Rectum* 10, 23–34. doi: 10.1007/BF02617382 6018347

[ref13] Fuloria S. Subramaniyan V. Sekar M. Wu Y. S. Chakravarthi S. Nordin R. B.. (2022). “Introduction to microbiome” in Microbiome in inflammatory lung diseases. eds. Gupta G. Oliver B. G. Dua K. Singh A. Mac Loughlin R. (Singapore: Springer Nature Singapore), 13–28.

[ref14] Furusawa Y. Obata Y. Fukuda S. Endo T. A. Nakato G. Takahashi D.. (2013). Commensal microbe-derived butyrate induces the differentiation of colonic regulatory T cells. Nature 504, 446–450. doi: 10.1038/nature12721, PMID: 24226770

[ref15] Garcia-Velasco J. A. Budding D. Campe H. Malfertheiner S. F. Hamamah S. Santjohanser C.. (2020). The reproductive microbiome—clinical practice recommendations for fertility specialists. Reprod. Biomed. Online 41, 443–453. doi: 10.1016/j.rbmo.2020.06.014, PMID: 32753361

[ref16] Garcia-Velasco J. A. Menabrito M. Catalan I. B. (2017). What fertility specialists should know about the vaginal microbiome: a review. Reprod. Biomed. Online 35, 103–112. doi: 10.1016/j.rbmo.2017.04.005, PMID: 28479120

[ref17] Garry Peterson C. R. A. Holling C. S. (1998). Ecological resilience, biodiversity, and scale. Ecosystems 1, 6–18. doi: 10.1007/s100219900002

[ref18] Genis S. Cerri R. L. A. Bach A. Silper B. F. Baylao M. Denis-Robichaud J.. (2018). Pre-calving intravaginal Administration of Lactic Acid Bacteria Reduces Metritis Prevalence and Regulates Blood Neutrophil Gene Expression after Calving in dairy cattle. Front. Vet. Sci. 5:135. doi: 10.3389/fvets.2018.00135, PMID: 29977896 PMC6021520

[ref19] Greenbaum S. Greenbaum G. Moran-Gilad J. Weintraub A. Y. (2019). Ecological dynamics of the vaginal microbiome in relation to health and disease. Am. J. Obstet. Gynecol. 220, 324–335. doi: 10.1016/j.ajog.2018.11.1089, PMID: 30447213

[ref20] Hickey R. J. Zhou X. Pierson J. D. Ravel J. Forney L. J. (2012). Understanding vaginal microbiome complexity from an ecological perspective. Transl. Res. 160, 267–282. doi: 10.1016/j.trsl.2012.02.008, PMID: 22683415 PMC3444549

[ref21] Huang B. Fettweis J. M. Brooks J. P. Jefferson K. K. Buck G. A. (2014). The changing landscape of the vaginal microbiome. Clin. Lab. Med. 34, 747–761. doi: 10.1016/j.cll.2014.08.006, PMID: 25439274 PMC4254509

[ref22] Human Microbiome Project Consortium (2012). Structure, function and diversity of the healthy human microbiome. Nature 486, 207–214. doi: 10.1038/nature11234, PMID: 22699609 PMC3564958

[ref23] Janda J. M. Abbott S. L. (2007). 16S rRNA gene sequencing for bacterial identification in the diagnostic laboratory: pluses, perils, and pitfalls. J. Clin. Microbiol. 45, 2761–2764. doi: 10.1128/JCM.01228-07, PMID: 17626177 PMC2045242

[ref25] Katharine Z. Coyte J. S. Foster K. R. (2015). The ecology of the microbiome: networks, competition, and stability. Science 350. doi: 10.1126/science.aad2602 26542567

[ref26] Koedooder R. Singer M. Schoenmakers S. Savelkoul P. H. M. Morre S. A. de Jonge J. D.. (2019). The vaginal microbiome as a predictor for outcome of in vitro fertilization with or without intracytoplasmic sperm injection: a prospective study. Hum. Reprod. 34, 1042–1054. doi: 10.1093/humrep/dez065 31119299

[ref27] Laguardia-Nascimento M. Branco K. M. Gasparini M. R. Giannattasio-Ferraz S. Leite L. R. Araujo F. M.. (2015). Vaginal microbiome characterization of Nellore cattle using metagenomic analysis. PLoS One 10:e0143294. doi: 10.1371/journal.pone.0143294, PMID: 26599789 PMC4657983

[ref28] Lamb G. C. Vitor C. D. Mercadante R. G. Bischoff K. (2014). What is the impact of infertility in beef cattle: UF IFAS Extension University of Florida.

[ref29] Lamont R. F. Sobel J. D. Akins R. A. Hassan S. S. Chaiworapongsa T. Kusanovic J. P.. (2011). The vaginal microbiome: new information about genital tract flora using molecular based techniques. BJOG 118, 533–549. doi: 10.1111/j.1471-0528.2010.02840.x, PMID: 21251190 PMC3055920

[ref30] Mageau R. C. A. M. (1999). What is a healthy ecosystem? Aquat. Ecol. 33, 105–115. doi: 10.1023/A:1009930313242

[ref31] Messman R. D. Contreras-Correa Z. E. Paz H. A. Perry G. Lemley C. O. (2020). Vaginal bacterial community composition and concentrations of estradiol at the time of artificial insemination in Brangus heifers. J. Anim. Sci. 98:skaa178. doi: 10.1093/jas/skaa178, PMID: 32515480 PMC7281872

[ref32] Miller E. A. Beasley D. E. Dunn R. R. Archie E. A. (2016). Lactobacilli dominance and vaginal pH: why is the human vaginal microbiome unique? Front. Microbiol. 7:1936. doi: 10.3389/fmicb.2016.01936 28008325 PMC5143676

[ref33] Miranda-Caso Luengo R. Lu J. Williams E. J. Miranda-Caso Luengo A. A. Carrington S. D. Evans A. C. O.. (2019). Delayed differentiation of vaginal and uterine microbiomes in dairy cows developing postpartum endometritis. PLoS One 14:e0200974. doi: 10.1371/journal.pone.0200974, PMID: 30629579 PMC6328119

[ref34] Moore S. G. Ericsson A. C. Poock S. E. Melendez P. Lucy M. C. (2017). Hot topic: 16S rRNA gene sequencing reveals the microbiome of the virgin and pregnant bovine uterus. J. Dairy Sci. 100, 4953–4960. doi: 10.3168/jds.2017-12592, PMID: 28434745 PMC6344888

[ref35] Petri R. M. Neubauer V. Humer E. Kroger I. Reisinger N. Zebeli Q. (2020). Feed additives differentially impact the Epimural microbiota and host epithelial gene expression of the bovine rumen fed diets rich in concentrates. Front. Microbiol. 11:119. doi: 10.3389/fmicb.2020.00119, PMID: 32140139 PMC7043141

[ref36] Quadros D. L. Zanella R. Bondan C. Zanella G. C. Facioli F. L. da Silva A. N.. (2020). Study of vaginal microbiota of Holstein cows submitted to an estrus synchronization protocol with the use of intravaginal progesterone device. Res. Vet. Sci. 131, 1–6. doi: 10.1016/j.rvsc.2020.03.027, PMID: 32278134

[ref37] Quereda J. J. García-Roselló E. Barba M. Mocé M. L. Gomis J. Jiménez-Trigos E.. (2020). Use of probiotics in intravaginal sponges in sheep: a pilot study. Animals 10:719. doi: 10.3390/ani10040719, PMID: 32326046 PMC7222760

[ref38] Ravel J. Gajer P. Abdo Z. Schneider G. M. Koenig S. S. McCulle S. L.. (2011). Vaginal microbiome of reproductive-age women. Proc. Natl. Acad. Sci. USA 108, 4680–4687. doi: 10.1073/pnas.1002611107, PMID: 20534435 PMC3063603

[ref39] Schmidt T. S. B. Raes J. Bork P. (2018). The human gut microbiome: from association to modulation. Cell 172, 1198–1215. doi: 10.1016/j.cell.2018.02.044 29522742

[ref40] Smith M. S. Hickman-Brown K. J. McAnally B. E. Oliveira Filho R. V. de Melo G. D. Pohler K. G.. (2023). Reproductive microbiome and cytokine profiles associated with fertility outcomes of postpartum beef cows. J. Anim. Sci. 101:skad 219. doi: 10.1093/jas/skad219 PMC10362934 37354343

[ref41] Smith S. B. Ravel J. (2017). The vaginal microbiota, host defence and reproductive physiology. J. Physiol. 595, 451–463. doi: 10.1113/JP271694, PMID: 27373840 PMC5233653

[ref42] Suez J. Zmora N. Zilberman-Schapira G. Mor U. Dori-Bachash M. Bashiardes S.. (2018). Post-antibiotic gut mucosal microbiome reconstitution is impaired by probiotics and improved by autologous FMT. Cell 174, 1406–1423 e 1416. doi: 10.1016/j.cell.2018.08.047 30193113

[ref43] Swartz J. D. Lachman M. Westveer K. O'Neill T. Geary T. Kott R. W.. (2014). Characterization of the vaginal microbiota of ewes and cows reveals a unique microbiota with low levels of lactobacilli and near-neutral pH. Front. Vet. Sci. 1:19. doi: 10.3389/fvets.2014.00019, PMID: 26664918 PMC4672155

[ref44] Taha T. E. Hoover D. R. Dallabetta G. A. Kumwenda N. I. Mtimavalye L. A. R. Yang L.-P.. (1998). Bacterial vaginosis and disturbances of vaginal flora: association with increased acquisition of HIV. AIDS 12, 1699–1706. doi: 10.1097/00002030-199813000-00019 9764791

[ref46] Turnbaugh P. J. Ley R. E. Mahowald M. A. Magrini V. Mardis E. R. Gordon J. I. (2006). An obesity-associated gut microbiome with increased capacity for energy harvest. Nature 444, 1027–1031. doi: 10.1038/nature05414, PMID: 17183312

[ref47] Walker B. (1995). Conserving biological diversity through ecosystem resilience. Conserv. Biol. 9, 747–752. doi: 10.1046/j.1523-1739.1995.09040747.x

[ref48] Wang Z. Klipfell E. Bennett B. J. Koeth R. Levison B. S. Dugar B.. (2011). Gut flora metabolism of phosphatidylcholine promotes cardiovascular disease. Nature 472, 57–63. doi: 10.1038/nature09922, PMID: 21475195 PMC3086762

[ref49] Wege Dias N. (2023). Changes in vaginal microbiome of beef cows enrolled in estrous synchronization protocols and its relation to fertility: Virginia Tech.

[ref50] Xu J. Bian G. Zheng M. Lu G. Chan W. Y. Li W.. (2020). Fertility factors affect the vaginal microbiome in women of reproductive age. Am. J. Reprod. Immunol. 83:e13220. doi: 10.1111/aji.13220, PMID: 31925865 PMC7078941

[ref51] Young V. B. (2017). The role of the microbiome in human health and disease: an introduction for clinicians. BMJ 356:j831. doi: 10.1136/bmj.j831, PMID: 28298355

